# Feasibility of Multimodal MRI-Based Deep Learning Prediction of High Amino Acid Uptake Regions and Survival in Patients With Glioblastoma

**DOI:** 10.3389/fneur.2019.01305

**Published:** 2019-12-17

**Authors:** Jeong-Won Jeong, Min-Hee Lee, Flóra John, Natasha L. Robinette, Alit J. Amit-Yousif, Geoffrey R. Barger, Sandeep Mittal, Csaba Juhász

**Affiliations:** ^1^Department of Pediatrics, Wayne State University School of Medicine and PET Center and Translational Imaging Laboratory, Children's Hospital of Michigan, Detroit, MI, United States; ^2^Department of Neurology, Wayne State University, Detroit, MI, United States; ^3^Translational Neuroscience Program, Wayne State University, Detroit, MI, United States; ^4^Department of Oncology, Wayne State University, Detroit, MI, United States; ^5^Karmanos Cancer Institute, Detroit, MI, United States; ^6^Department of Neurosurgery, Wayne State University, Detroit, MI, United States; ^7^Virginia Tech Carilion School of Medicine and Carilion Clinic, Roanoke, VA, United States

**Keywords:** glioblastoma, multimodal MRI, positron emission tomography, amino acid, tryptophan, deep learning

## Abstract

**Purpose:** Amino acid PET has shown high accuracy for the diagnosis and prognostication of malignant gliomas, however, this imaging modality is not widely available in clinical practice. This study explores a novel end-to-end deep learning framework (“U-Net”) for its feasibility to detect high amino acid uptake glioblastoma regions (i.e., metabolic tumor volume) using clinical multimodal MRI sequences.

**Methods:** T2, fluid-attenuated inversion recovery (FLAIR), apparent diffusion coefficient map, contrast-enhanced T1, and alpha-[^11^C]-methyl-L-tryptophan (AMT)-PET images were analyzed in 21 patients with newly-diagnosed glioblastoma. U-Net system with data augmentation was implemented to deeply learn non-linear voxel-wise relationships between intensities of multimodal MRI as the input and metabolic tumor volume from AMT-PET as the output. The accuracy of the MRI- and PET-based volume measures to predict progression-free survival was tested.

**Results:** In the augmented dataset using all four MRI modalities to investigate the upper limit of U-Net accuracy in the full study cohort, U-Net achieved high accuracy (sensitivity/specificity/positive predictive value [PPV]/negative predictive value [NPV]: 0.85/1.00/0.81/1.00, respectively) to predict PET-defined tumor volumes. Exclusion of FLAIR from the MRI input set had a strong negative effect on sensitivity (0.60). In repeated hold out validation in randomly selected subjects, specificity and NPV remained high (1.00), but mean sensitivity (0.62), and PPV (0.68) were moderate. AMT-PET-learned MRI tumor volume from this U-net model within the contrast-enhancing volume predicted 6-month progression-free survival with 0.86/0.63 sensitivity/specificity.

**Conclusions:** These data indicate the feasibility of PET-based deep learning for enhanced pretreatment glioblastoma delineation and prognostication by clinical multimodal MRI.

## Introduction

Glioblastomas are the deadliest primary brain tumors, and their initial treatment (surgery followed by radiation), based on clinical MRI, can miss tumor portions infiltrating to adjacent brain regions. Accurate non-invasive imaging of tumor-infiltrating brain is critical to optimize surgical resection and subsequent radiation therapy and prolong survival (1, 2). However, current clinical MRI, including T1-weighted images with gadolinium (T1-Gad), T2, and fluid-attenuated inversion recovery (FLAIR), has limited accuracy to detect such infiltrating regions and predict survival, since they cannot accurately differentiate regions with active tumor from vasogenic edema and necrosis (3).

To overcome the limitations of conventional MRI, advanced imaging techniques, including perfusion MRI (3, 4), diffusion-weighted imaging (DWI) (5, 6), and positron emission tomography (PET) (7–9) are being actively investigated. Our previous studies reported that high amino acid uptake measured by alpha-[^11^C]-methyl-L-tryptophan (AMT)-PET can accurately detect both enhancing and non-enhancing gliomas (10–13). AMT-PET can estimate amino acid transport and tryptophan metabolism via the immunosuppressive kynurenine pathway (12). Increased AMT uptake often extends beyond the contrast-enhancing tumor to identify glioma-infiltrated brain (10), which is commonly underestimated based on clinical MRI. High AMT uptake also has a strong prognostic value for survival in patients with recurrent high-grade glioma (14). However, AMT-PET cannot gain widespread clinical use due to the short half-life (20 min) of ^11^C, limiting its clinical use to institutions equipped with an on-site cyclotron. Although other, ^18^F-labeled amino acid PET tracers are more widely available, their use is still confined to a limited number of centers worldwide.

This study explores a novel end-to-end deep learning network to test its ability to detect high tryptophan uptake glioblastoma regions (i.e., the metabolic tumor volume) using clinical multimodal MRI. Deep learning network, specifically U-Net (15–17), is a powerful computational approach capable of automatically learning non-linear relationships between features and patterns existing in multimodal images. This approach showed promise lately in performing automatic lesion detection by amino acid PET in gliomas (18) with manually segmented PET tumor volume as the ground truth. However, the use of multimodal MRI to delineate tumor areas with high amino acid uptake has not been evaluated, and the prognostic value of the deep learning methods have yet to be investigated in depth.

In this study we investigated whether deep learning can mine complex relationships between intensities on multimodal MRI in PET-determined glioblastoma volumes, i.e., areas of high AMT uptake used as the ground truth. The primary goal was to evaluate if the advanced deep learning technique could be translated to clinical practice, where AMT-PET (and other amino acid PET) is unavailable in most centers, using multimodal MRI to demarcate the PET-defined boundaries of infiltrating gliomas and improve survival prediction. Our central hypothesis was that tumor volume extracted from multimodal MRI via deep learning can predict the metabolic tumor volume determined by PET. We also explored if the deep learning-based glioma volume is more accurate to predict progression-free survival (PFS) than the contrast-enhancing tumor volume. To determine the efficacy of the proposed U-Net approach across different MRI scanners and imaging parameters, we also compared MRI data acquired using routine protocols on two different 3T MRI scanners.

## Materials and Methods

### Subjects

Patients were selected retrospectively from a single-center PET database of 73 adult subjects with newly-diagnosed glioma (WHO grade I-IV) who underwent pre-treatment AMT-PET scanning at the PET Center, Children's Hospital of Michigan between February 1, 2008 and January 31, 2018. The final study group included 21 patients (age: 58 ± 12 years, 12 males; Supplementary Table 1), who met the following inclusion criteria: (i) histopathologically-verified glioblastoma (WHO grade IV), (ii) available complete pre-treatment multimodal MRI data set including non-contrast T2/FLAIR, DWI, and T1-Gad acquired on one of two 3T MRI scanners (see details below). Twenty patients had tumor resection and subsequent chemoradiation; in one patient (#10), who died of pulmonary embolism before surgery, glioblastoma was diagnosed by autopsy. Clinical outcomes included PFS (not including the patient who died before treatment), determined by serial MRI and clinical follow-up. The study was approved by Wayne State University's Institutional Review Board, and written informed consent was obtained from all participants in accordance with the Declaration of Helsinki.

### Data Acquisition and Preparation

Multimodal MRI protocols were applied in each patient on one of two different 3T scanners (see acquisition parameters for Siemens Protocol [Siemens Trio] and Philips Protocol [Philips Achieva] in Supplementary Table 2). These were utilized to assess the effect of specific MRI protocols on the performance of the U-Net analysis. The contrast-enhancing tumor volume was measured semiautomatically using 3D Slicer, as described previously (10). This procedure was repeated by the same investigator (FJ) on different days to establish reproducibility of the volume measures. All patients underwent the same pre-treatment AMT-PET scanning protocol (10, 13, 14) using a GE Discovery STE PET/CT scanner with a median interval of 3 days between the MRI and PET/CT scans. Briefly, after 6-h fasting, AMT (37 MBq/kg) was injected via a venous line. At 25 min after AMT injection, a dynamic emission scan of the brain (7 × 5 min) was acquired. Measured attenuation correction, scatter, and decay correction were applied to all images. For visualization of AMT uptake, averaged activity images 30–55 min post-injection were created and converted to an AMT standardized uptake value (SUV) image.

The actual intensities of multimodal MR images were scaled by the global mean. A binary mask of the metabolically active tumor: P(x), was obtained as the ground truth from AMT-PET by applying a previously established threshold of 1.65 tumor/normal cortex ratio of AMT SUV (13, 14). All images (including ground truth binary PET mask) were spatially co-registered and resampled at the same resolution (1 × 1 × 1 mm) and matrix volume (240 × 240 × 150). Each multimodal volume was normalized from mean and standard deviation. Single data-augmentation scheme was applied to enlarge the training dataset up to 100 augmentations per patient. This procedure was iteratively performed by applying an arbitrary affine transformation to multimodal MRI data of individual patients.

### U-Net Construction and Implementation

The detailed U-Net system architecture (15) is shown on Supplementary Figure 1. U-Net consists of an encoding (or collapsing) path which took a series of input slice images: T1-Gad, T2, FLAIR, ADC, and a decoding (or expanding) path, which returned a binary slice image as an output: AMT-PET-learned MRI-based tumor volume, PM(x) (0: non-tumor, 1: tumor).

To investigate the effect of different multimodal MRI protocols on the performance of the U-Net system, three different U-Net systems (U-Net_1(Siemens)_ trained by multimodal data of Siemens Protocol, U-Net_2(Philips)_ trained by multimodal data of Philips Protocol, and U-Net_3_ trained by combined multimodal [Siemens+Philips] Protocol) were separately implemented using Google TensorFlow library (www.tensorflow.org). Each U-Net system was designated to deeply learn non-linear voxel-wise relationships between “given input: multimodal MRI data” and “targeted output: AMT-PET tumor mask [i.e., P(x)],” where dice similarity coefficient (DSC) was used as a measure of detectability and maximized by back-propagating a loss function (i.e., minus DSC) through the Adam optimizer (19). Batch size and learning rate were set to 16 and 10^−3^, respectively. The augmented dataset was randomly divided into training and testing data (70 vs. 30%, respectively) to evaluate the convergence of the three U-Net systems in the original augmented dataset to investigate the upper limit of U-Net accuracy in the selected study cohort (*n* = 21). In addition, we applied a repeated hold-out validation procedure to evaluate the performance of the proposed U-Net in predicting target: P(x) from untrained subsets, U-Net_4_, where the augmented data of 17 subjects were randomly assigned to train and test the convergence of U-Net_4_, and the converged U-Net_4_ was then used to predict P(x) of the remaining 4 subjects for validation. We performed the above validations in randomly assigned 17/4 subjects (training/testing), repeated 100 times, to evaluate the overall performance of U-Net_4_.

To determine which MRI modality had the strongest influence on the prediction of the PET-defined tumor region, four different three-channel U-Net_3_ systems were evaluated to learn P(x) from combinations of input MRI modalities, (1) [T1-Gad, FLAIR, ADC], (2) [T2, FLAIR, ADC], (3) [T1-Gad, T2, FLAIR], and (4) [T1-Gad, T2, ADC]. The performance of each three-channel U-Net_3_ system was compared to that of four-channel U-Net_3_ system which learned P(x) from all four MRI modalities (baseline condition) to determine which three-channel U-Net system has the greatest decrease of sensitivity from baseline condition, suggesting that an MRI modality excluded in that three-channel U-Net_3_ system is the most influential for predicting P(x).

### Statistical Analysis

The reproducibility of the T1-Gad enhancing volume, M(x), measured by a semiautomatic method was tested by intra-class correlation (ICC).

Sensitivity, specificity, positive predictive value (PPV), and negative predictive value (NPV) were evaluated between “output: PM(x)” and “target: P(x)”. To test how well U-Net_4_-based PET-learned MRI tumor volumes can approximate the original PET-based tumor volumes, corresponding volumes were correlated using Pearson's correlations. To test if P(x) and/or PM(x) outperform M(x) for predicting PFS, a survival analysis was performed with the following image-derived tumor volumes as predictors: (1) P^+^: AMT-PET tumor mask volume, (2) M^+^: contrast-enhancing tumor volume from the T1-Gad image, and the following U-Net_4_-based volumes: (3) PM^+^: AMT-PET-learned MRI-based tumor volume, (4) PM^+^M^+^: total tumor voxel volume in PM(x) ⋂ M(x) (5) PM^+^M^−^: total AMT-PET-learned MRI-based tumor voxel volume in PM(x) ⋂ M^c^ (i.e., outside the contrast-enhancing glioblastoma volume, consistent with tumor-infiltrated non-enhancing brain, and (6) PM^−^M^+^: total tumor voxel volume in PM^c^(x) ⋂ M(x), consistent with enhancing tumor volume without high metabolic activity, a glioblastoma subregion recently reported by our group (13).

For each volume measure as well as non-binary prognostic variables (age, Karnofsky Performance Status [KPS] scores, Ki-67 tumor proliferative index), a receiver operating characteristic (ROC) analysis was performed to determine an area under the curve (AUC), and optimal sensitivity and specificity was determined for 6-month PFS. Binary prognostic variables (MGMT promoter methylation status, IDH1 mutation status, and resection extent [partial vs. gross total]) were entered in a Cox regression analysis to identify non-imaging predictors of PFS. A *p*-value of < 0.05 was considered to be significant.

## Results

The intra-observer reproducibility of the T1-Gad tumor volume measurement was strong with an ICC of 0.988 (*p* < 0.001). The T1-Gad volumes extracted on two different measurements were similar (mean: 18.51 ± 1.50 cm^3^ vs. 18.57±1.39 cm^3^). Thus, the average volume of the two T1-Gad volume measurements was used as M(x) in further analyses.

### Comparison of PET-Defined and U-Net-Learned MRI-Defined Tumor Volumes

After 5000 epochs, DSC values of the three U-Net systems to predict target: P(x), U-Net_1(Siemens)_/U-Net_2(Philips)_/U-Net_3_, reached 0.98(0.98)/0.99(0.99)/0.98(0.98) in the training (testing) set, respectively. At the voxel level, the resulting three U-Net systems led to ≥0.85 sensitivity, 1.00 specificity, ≥0.81 PPV, and 1.00 NPV (detailed values are listed in Supplementary Table 3). In the repeated hold-out validation, U-Net_4_ achieved 0.62 ± 0.23 sensitivity, 1.00 ± 0.00 specificity, 0.68 ± 0.19 PPV, and 1.00 ± 0.00 NPV (Supplementary Table 3), suggesting that the proposed U-Net_4_ can achieve about 84% of the U-Net_3_ PPV by correctly predicting P(x) from an independent patient cohort not included in both training and testing procedures of the U-Net_4_. The AMT-PET-learned MRI tumor volume (PM^+^) from U-Net_4_ showed a positive correlation with the AMT-PET tumor volume P(x) (r = 0.61, p = 0.03). P(x) showed an even stronger correlation with PM^+^M^+^, i.e., the U-Net_4_-based PET-learned tumor volume inside the contrast-enhancing MRI volume (*r* = 0.75, *p* < 0.001).

### Comparison of the Effect of Different MRI Protocols and Specific MRI Sequences on Metabolic Tumor Volume Prediction

**Figure 1:**
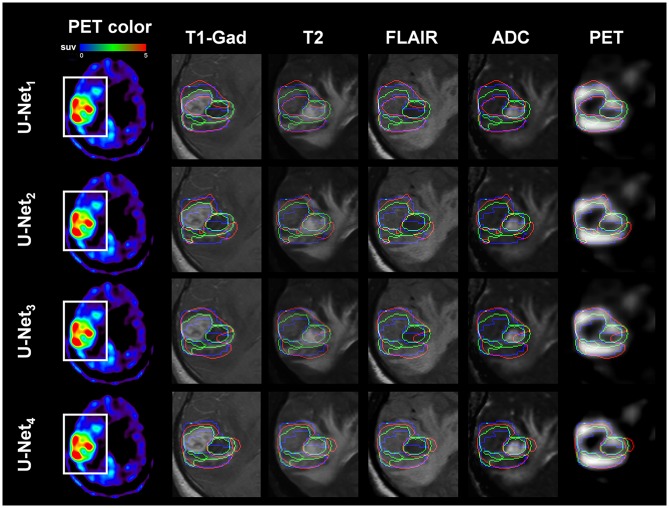
Representative example of AMT-PET-learned MRI-based tumor volume: PM(x) (voxels inside red contour), where multi-modal MRI data of Patient No. 9 (images acquired with Siemens Protocol) were separately analyzed by U-Net_1(Siemens)_ (1st row), U-Net_2(Philips)_ (2nd row), U-Net_3_ (3rd row), and U-Net_4_ (4th row). Note that U-Net_1(Siemens)_ and U-Net_3_ learned multi-modal MRI of Patient 9 and outperformed the other two U-Net systems to spatially match PM(x) with the target, AMT-PET tumor volume: P(x) (voxels inside blue contour); sensitivity/specificity/ PPV/NPV = 0.86/1.00/0.82/1.00, 0.41/1.00/0.51/1.00, 0.85/0.89/0.69/1.00, and 0.70/1.00/0.74/1.00 for U-Net_1(Siemens)_, U-Net_2(Philips)_, U-Net_3_ and U-Net_4_, respectively. For comparison, T1-Gad tumor volume: M(x) (voxels inside green contour) was superimposed. White box indicates the region of interest where T1-Gad, T2, FLAIR, ADC map, and AMT-PET slices were captured to show the contours.

**Figure 2:**
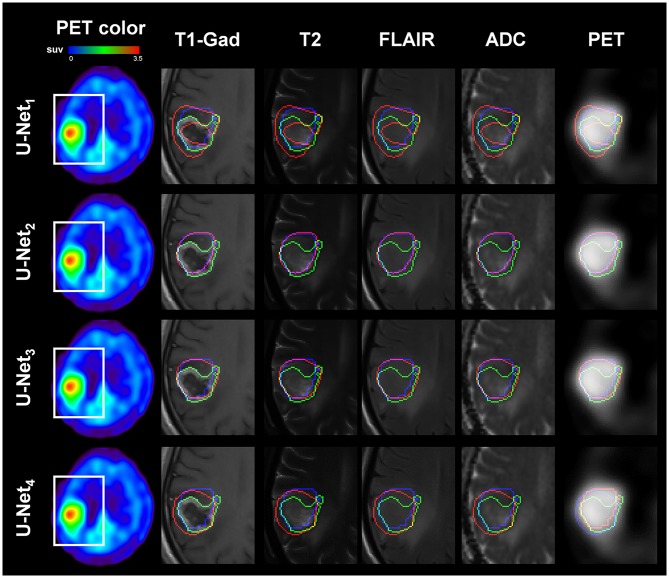
Representative example of AMT PET-learned MRI-based tumor volume: PM(x) (voxels inside red contour) where multi-modal MRI data of Patient No. 13 (images acquired with Philips Protocol) were separately analyzed by U-Net_1(Siemens)_ (1st row), U-Net_2(Philips)_ (2nd row), U-Net_3_ (3rd row), and U-Net_4_ (4th row). U-Net_2(Philips)_ and U-Net_3_ learned multi-modal MRI of Patient 13 and outperformed the other two U-Net systems to spatially match P(x) with the target, AMT-PET tumor volume: P(x) (blue contour), sensitivity/specificity/PPV/NPV = 0.65/1.00/0.40/1.00, 0.95/1.00/0.79/1.00, 0.94/1.00/0.75/1.00, and 0.91/1.00/0.68/1.00 for U-Net_1(Siemens)_, U-Net_2(Philips)_, U-Net_3_ and U-Net_4_, respectively. For comparison, T1-Gad tumor volume: M(x) (voxels inside green contour) was superimposed. White box indicates the region of interest where T1-Gad, T2, FLAIR, ADC map, and AMT-PET slices were captured to show the contours.

Figures 1, 2 present representative examples to predict the AMT-PET-learned MRI-based tumor volume, PM(x), using the proposed U-Net systems with multimodal MRI data acquired using two different clinical MRI protocols. Both U-Net_3_ and U-Net_4_, which fully learned non-linear relationships of multimodal MRI data, provided the PET-learned tumor volumes [PM(x)], which were spatially well-matched with the PET-based ground truth, P(x), indicating that systematic learning of different MRI protocol data can supplement conventional MRI by approximating glioblastoma volume with high amino acid uptake. In addition, PM(x) of U-Net_3_ (mean: 41.5 ± 30.1 cm^3^) and U-Net_4_ (mean: 31.0 ± 22.5 cm^3^) were correlated (*R* = 0.50, *p* = 0.02), and both were significantly larger than M^+^, which indicates voxels of T1-Gad enhancement (mean: 18.5 ± 14.4 cm^3^) at *p* < 0.01 in a paired *t*-test.

Among the four MRI sequences, exclusion of FLAIR images yielded the greatest decrease in sensitivity to detect true positive voxels of P(x), as compared with the exclusion of other three MRI modalities (sensitivity of four-channel U-Net_3_ = 0.85, sensitivity of three-channel U-Net_3_ with [T1-Gad, FLAIR, ADC]/[T2, FLAIR, ADC]/[T1-Gad, T2, FLAIR]/[T1-Gad, T2, ADC] = 0.84/0.79/0.81/0.60). Specificity remained at 1.0 with all three-channel U-Net_3_ systems.

### Survival Prediction

Mean PFS (data available in 17 patients) was 232 days (7.7 months). The results of the ROC analysis with U-Net_4_-based tumor volumes for 6-month PFS prediction are shown in Table 1. The highest AUC (0.66) was achieved by PM^+^⋂ M^+^ with a sensitivity/specificity of 0.86/0.63. In contrast, the contrast-enhancing volume, M^+^, had a lower AUC of 0.45, with sensitivity of 0.71 and specificity of only 0.38 to predict 6-month PFS. None of the clinical or molecular prognostic variables predicted PFS.

**Table 1:** Results of the receiver operating characteristic analysis to predict 6-month progression-free survival by imaging (MRI, PET, and MRI-learned PET from U-Net_4_) and clinical variables.

**Prognostic Variables**	**AUC**	**95% CI**	**Sensitivity**	**Specificity**
T1-Gad volume: M^+^	0.45	0.14–0.75	0.71	0.38
AMT-PET tumor volume: P^+^	0.69	0.41–0.98	0.75	0.78
AMT-PET-learned MRI tumor volume: PM^+^	0.43	0.12–0.74	0.57	0.38
Volume combinations: PM^+^M^+^	0.66	0.37–0.95	0.86	0.63
PM^+^M^−^	0.32	0.04–0.61	0.71	0.25
PM^−^M^+^	0.64	0.35–0.93	0.71	0.63
**Clinical**				
Age	0.19	0.0–0.41	0.62	0.22
KPS score	0.52	0.21–0.83	0.50	0.33
Ki-67 proliferative index	0.28	0.02–0.53	0.75	0.22

*KPS, Karnofsky Performance Status*.

## Discussion

This study provides proof-of-concept results that PET-learned MRI-based tumor volumes can approximate metabolic tumor volumes defined by amino-acid PET imaging. Thus, the results suggest that advanced deep learning can be translated to clinical practice where AMT-PET is currently unavailable and the use of other amino acid PET tracers is also limited. The data also suggest that the intersection of PET-learned MRI-based tumor volume and the contrast-enhancing tumor volume may provide better prognostic information than the standalone contrast-enhancing tumor volume. These results will need to be tested in future studies in independent data sets and with other amino acid PET tracers used as the ground truth for tumor metabolic volume.

To the best of our knowledge, this study is the first to demonstrate that advanced U-Net may enhance detection of the metabolic tumor volume of glioblastoma by PET-learned multi-modal MRI acquired in the clinical setting, where amino acid PET is often not available to detect non-enhancing infiltrative areas. Such regions often show MRI signal abnormalities on FLAIR, T2, or DWI, and the three-channel, leave-one-out analysis suggested that the FLAIR sequence may have the strongest influence on the prognostic value of the full four-channel U-Net system on the metabolic tumor volume. Active glioma contrast on FLAIR images may be the most similar to the PET-derived volume and provides the most effective low-level intensity features to the U-Net convolution layers. Non-enhancing FLAIR-positive tumor regions often encompass glioma-infiltrated brain can be underestimated and undertreated in clinical practice, and, therefore, they are high-risk areas for post-treatment tumor progression (13). A recent study indeed showed that pre-treatment MRI volumes, including both enhancing and FLAIR-based volumes, are poor predictors of PFS, particularly in patients with gross total glioblastoma resections (20). Future studies could incorporate additional MRI-based volume measurements for comparisons in a more comprehensive analysis.

Several studies have attempted to predict survival from multimodal MRI data by mining radiomic features via classical machine learning approaches (21–24) and deep learning models (25, 26). All these studies differ methodologically and typically require heavy computational loads for evaluating and ranking a huge number of features, which led to mixed outcomes depending on the employed algorithm, the types of the MRI sequences, and analysis work-flow. The main innovation of this study is the use of a deep learning approach called U-Net to directly detect metabolically active (PET-defined) glioma volume from multimodal MRI by learning intricate and abstract non-linear relationships of MRI intensities in active glioma regions showing high AMT uptake. The resulting U-Net system can identify metabolically active glioma in a set of multimodal MRI data according to the learned non-linear relationships across voxels.

This study has some limitations. First, the limited sample size can be problematic for individual U-Net to learn heterogeneous multimodal feature maps via deep learning process (27). Although data augmentation was applied to alleviate this limitation, it included all subjects' data for augmenting training/testing instances based on affine transformation simulating spatially variant glioblastoma (18, 28), which may cause overfitting of an individual U-Net, since the intensities of multimodal MRI having different resolutions are over-sampled at isotropic grids. We presumed that the use of the original augmented dataset helps us investigate the upper limit of the U-Net accuracy for detection of spatially variant glioblastomas in the selected study cohort. The high detection accuracy provided by Unet_1−3_ is likely an overestimation due to heavy data augmentation applied due to the limited sample size. The reproducibility and real-life accuracy of each U-Net should be evaluated at a new, independent dataset obtained from other advanced data augmentation strategies (29). Our hold-out validation analysis in U-Net_4_ with a subgroup of our patients showed the feasibility of this latter approach, but, as expected, it showed lower accuracy values. The true accuracy of this approach will need to be tested in a larger, independent patient population. This could be feasible with the use of more commonly utilized amino acid PET tracers (such as O-(2-[^18^F]fluoroethyl)-L-tyrosine) (30). Finally, known molecular genetic prognostic markers (such as IDH mutation and MGMT promoter methylation) were not associated with survival in this small cohort, and the limited sample size precluded multivariate survival analyses. This may have contributed to the lack of prognostic value of the imaging variables for overall survival.

## Conclusions

After further validation in larger, independent cohorts, the tested U-Net approach may be useful in the presurgical evaluation of glioblastoma by supplementing conventional multimodal MRI to approximate glioblastoma volume with high amino acid uptake. The benefits of the proposed approach include the lack of added risk or cost to improve tumor delineation and survival prediction using clinically acquired multimodal MRI, and fully automated end-to-end analysis pipeline that does not require subjective and complex intervention in raw MRI data. Future studies could expand this deep learning application to assisting evaluation of gliomas with different grades and detecting post-treatment glioma recurrence.

## Data Availability Statement

The datasets generated during and/or analyzed during the current study are available from the corresponding author on reasonable request.

## Ethics Statement

The studies involving human participants were reviewed and approved by Wayne State University IRB. The patients/participants provided their written informed consent to participate in this study.

## Author Contributions

J-WJ, SM, and CJ conceptualized and designed the study. FJ, NR, AA-Y, GB, SM, and CJ were involved in data collection including MRI, PET, and clinical data collections. M-HL and FJ carried out data analysis, including initial multimodal image processing and co-registration. J-WJ and M-HL performed the deep learning analysis. FJ and CJ performed the survival analyses. J-WJ and M-HL drafted and revised the manuscript with the intellectual inputs from FJ, NR, AA-Y, GB, SM, and CJ.

### Conflict of Interest

The authors declare that the research was conducted in the absence of any commercial or financial relationships that could be construed as a potential conflict of interest.
