# Supplementary material for: Feasibility of Multimodal MRI-Based Deep Learning Prediction of High Amino Acid Uptake Regions and Survival in Patients With Glioblastoma
Source: Front Neurol. 2019 Dec 17;10:1305. doi: 10.3389/fneur.2019.01305 (PMC6928045; doi:10.3389/fneur.2019.01305)
Supplement: Supplementary file 1 [file Table_1.docx]

**Supplementary figure 1.** A diagram of the four-layer U-Net architecture. Each box corresponds to a multi-modal feature map, where the size is shown at the lower edge of the box. The gray arrows denote copy and crop operation. The presented U-Net system is a popular network consisting of an encoding (or collapsing) path which takes a series of input slice images from four multi-modal MRI sequences: T1-Gad, T2, FLAIR, ADC, and a decoding (or expanding) path, which returns a binary slice image as an output: AMT-PET-learned MRI-based tumor volume: PM(x) (i.e., 0: non-tumor, 1: tumor). The encoding path gradually reduces the spatial dimension of input data with repeated application of two 3×3 convolution (Conv), batch normalization (BN), rectified linear unit (ReLU), and 2×2 maxPooling operation for downsampling. A decoding gradually recovers the object details and spatial dimension where each step has an upsampling of feature map with a 3×3 deconvolution (Decon), a concatenation with the correspondingly cropped feature map from encoding path, and a 3×3 convolution, followed by a BN and a ReLu. At the final layer, a 1×1 convolution is applied to map each 8-component feature vector to the desired number of classes (i.e., 0: non-tumor, 1: tumor). Our implementation has 19 blocks to be trained by fully learning nonlinear relationships between multi-modal features and use the fully learned layers to make a binary voxel-wise decision in PM(x), either tumor (1) or non-tumor (0), at every voxel of a given multi-modal MRI data.


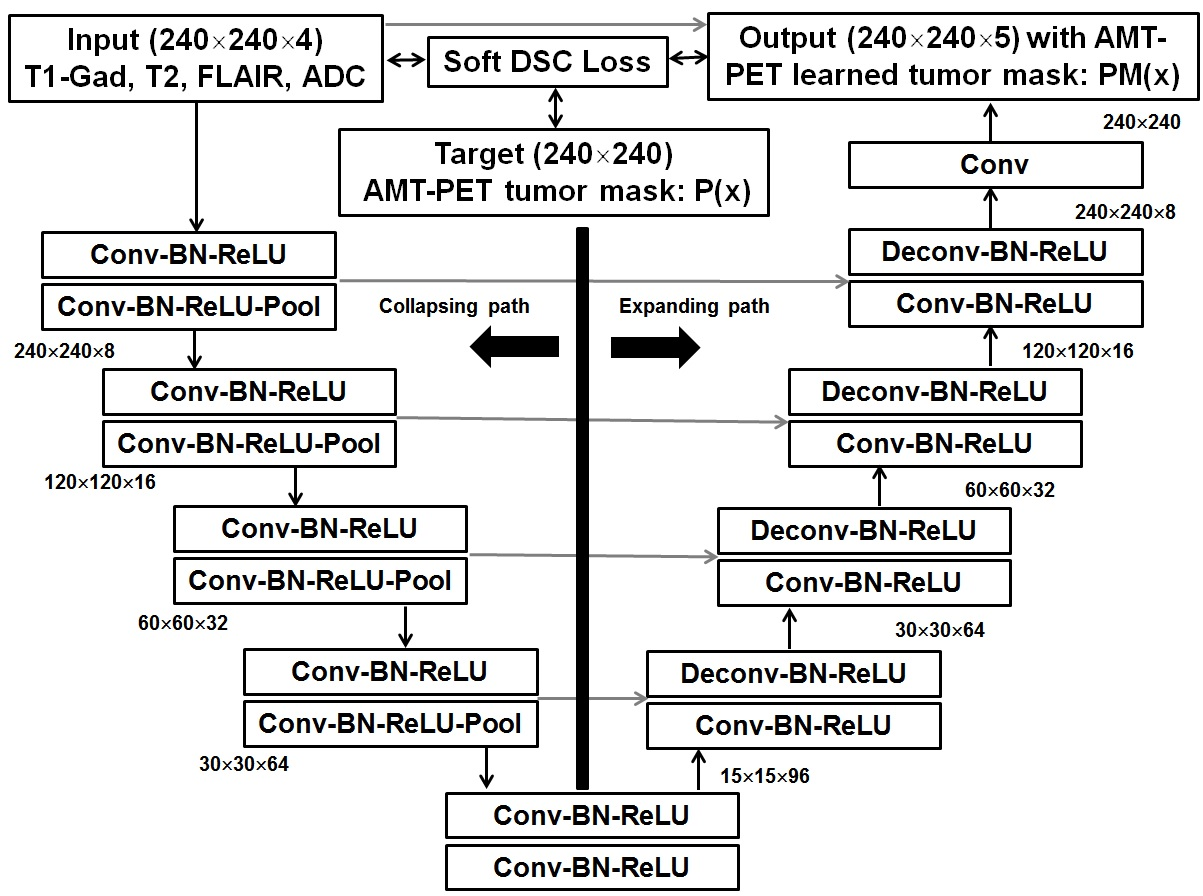


**Supplementary table 1.** Clinical data and prognostic glioma markers of the 21 study patients.

| Patient No. | Gender | Age  (years) | KPS  score | Resection type | Ki-67  (%) | IDH1 | MGMT  methylation | PFS  (days) |
| --- | --- | --- | --- | --- | --- | --- | --- | --- |
| 1 | M | 51 | 100 | total | 20 | wild | no | 105 |
| 2 | M | 24 | 80 | subtotal | 20-25 | wild | no | 5 |
| 3 | F | 65 | 40 | total | 10-15 | wild | no | N/A |
| 4 | M | 53 | 80 | total | 10 | wild | no | 92 |
| 5 | M | 67 | 80 | total | 25 | wild | no | 220 |
| 6 | M | 66 | 90 | subtotal | 25-30 | wild | yes | 630 |
| 7 | F | 51 | 100 | total | 20 | wild | no | N/A |
| 8 | F | 60 | 90 | total | >50 | mutated | yes | 726 |
| 9 | M | 62 | 90 | total | 5-30 | wild | no | 217 |
| 10 | M | 74 | 90 | N/A | N/A | wild | yes | N/A |
| 11 | F | 52 | 80 | total | 30-40 | mutated | no | 200 |
| 12 | F | 63 | 90 | total | 30-40 | wild | no | 87 |
| 13 | M | 46 | 80 | total | 25 | wild | no | 91 |
| 14 | F | 56 | 90 | subtotal | 35-40 | wild | yes | 226 |
| 15 | M | 47 | 90 | subtotal | 10-15 | wild | no | 508 |
| 16 | F | 70 | 50 | subtotal | >50 | wild | no | N/A |
| 17 | M | 79 | 90 | total | 30 | wild | yes | 254 |
| 18 | F | 54 | 100 | total | 25-30 | wild | yes | 133 |
| 19 | M | 54 | 100 | subtotal | 15-20 | wild | no | 88 |
| 20 | F | 53 | 60 | total | 20-25 | wild | yes | 86 |
| 21 | M | 65 | 80 | total | 15-50 | wild | yes | 274 |

Patients #1-12: Siemens MRI Protocol; Patients #13-21: Philips MRI Protocol. *: Patient was alive at last follow-up; ^#^: Tumor histology established at autopsy.

Abbreviations: M: male; F: female; KPS: Karnofsky Performance Status; N/A: not available; IDH1: isocitrate dehydrogenase 1; MGMT: O-(6)-methylguanine-DNA methyltransferase; PFS: progression-free survival

**Supplementary table 2**. Multimodal MRI protocols of the two scanners used for this project.

|  | **Protocol 1** | **Protocol 2** |
| --- | --- | --- |
|  | Siemens Trio 3T | Philips Achieva 3T |
| T1-Gad  TR/TE/TI/voxel size | SE IR Axial  2500/12/1100/0.45×0.45×4 mm^3^ | SE SS Axial  508/10/Not applied/0.45×0.45×4 mm^3^ |
| T2  TR/TE/voxel size | TSE FS BLADE Axial  5000/113/0.72×0.72×5 mm^3^ | TSE SPAIR Axial  3000/80/0.44×0.44×4 mm^3^ |
| FLAIR  TR/TE/TI/voxel size | FLAIR BLADE Axial  9000/109/2500/0.72×0.72×4 mm^3^ | FLAIR CLEAR Axial  11000/125/2800/0.60×0.60×4 mm^3^ |
| DWI  TR/TE/voxel size | EP2D Diff Axial  5000/78/1.80×1.80×4 mm^3^ | isoDiffusion Axial  3140/70/1.87×1.87×4 mm^3^ |

T1-Gad: post-gadolinium T1; FLAIR: fluid-attenuated inversion recovery; DWI: diffusion-weighted imaging; TR: repetition time (ms); TE: echo time (ms); TI: inversion time (ms); TSE: turbo spin echo; FS: fast spin; BLADE: Siemens’ proprietary name for a radial sampling method using rotating blades composed of multiple phase-encoded lines; SPAIR: spectral attenuated inversion recovery

**Supplementary table 3.** Overall performance of individual U-Net systems to detect AMT-PET tumor volume from multi-modal MRI data. Representative cases are shown on Figures 1 and 2.

| Model | | U-Net_1_ | U-Net_2_ | U-Net_3_ | U-Net_4_ |
| --- | --- | --- | --- | --- | --- |
| Data | | Siemens Protocol | Philips Protocol | Protocols 1 and 2 | |
| Number of augmented data | | 1200 | 900 | 2100 | |
| DSC (training/testing) | | 0.98/0.98 | 0.99/0.99 | 0.98/0.98 | 0.79*/0.68* |
| Sensitivity | mean±SD | 0.87±0.05 | 0.92±0.05 | 0.85±0.08 | 0.62±0.23 |
|  | max/min | 0.95/0.79 | 0.96/0.80 | 0.96/0.69 | 0.91/0.15 |
|  | 95% CI | 0.83-0.90 | 0.88-0.95 | 0.82-0.89 | 0.49-0.76 |
| Specificity | mean±SD | 1.00±0.00 | 1.00±0.00 | 1.00±0.00 | 1.00±0.00 |
|  | max/min | 1.0/1.0 | 1.00/1.00 | 1.00/1.00 | 1.00/1.00 |
|  | 95% CI | 1.00-1.00 | 1.00-1.00 | 1.00-1.00 | 1.00-1.00 |
| PPV | mean±SD | 0.86±0.06 | 0.84±0.04 | 0.81±0.08 | 0.68±0.19 |
|  | max/min | 0.95/0.78 | 0.89/0.79 | 0.96/0.70 | 1.00/0.28 |
|  | 95% CI | 0.83-0.90 | 0.81-0.87 | 0.77-0.85 | 0.60-0.76 |
| NPV | mean±SD | 1.00±0.00 | 1.00±0.00 | 1.00±0.00 | 1.00±0.00 |
|  | max/min | 1.00/1.00 | 1.00/1.00 | 1.00/1.00 | 1.00/0.99 |
|  | 95% CI | 1.00-1.00 | 1.00-1.00 | 1.00-1.00 | 1.00-1.00 |

DSC: dice similarity coefficient; SD: standard deviation; CI: confidence interval; PPV: positive predictive value; NPV: negative predictive value. *: average value from 100 random trials of 17 training/4 test subjects in the repeated hold-out validation.
